# The Association of Neuropathic Pain with Function, Disease Severity, Emotional Status, Sleep Disturbance, and Quality of Life in Patients with Knee Osteoarthritis

**DOI:** 10.3390/jcm15176850

**Published:** 2026-09-04

**Authors:** Vijdan Yıldız Özkul, Münevver Serdaroğlu Beyazal, Gül Devrimsel, Murat Yıldırım

**Affiliations:** 1 Department of Physical Medicine and Rehabilitation, Erzurum City Hospital, Erzurum 25240, Türkiye; 2 Department of Physical Medicine and Rehabilitation, Faculty of Medicine, Recep Tayyip Erdoğan University, Rize 53100, Türkiye

**Keywords:** osteoarthritis, knee, pain, neuropathic pain

## Abstract

**Background/Objectives:** The present study aimed to evaluate the frequency of neuropathic-like pain (NP) in patients with symptomatic knee osteoarthritis (KOA) and its association with function, disease severity, emotional status, sleep disturbance, and quality of life. **Methods:** Consecutive patients with symptomatic KOA were included in this cross-sectional study. NP was evaluated using the painDETECT Questionnaire (PD-Q) and DN4. Functional status, disease severity, depression, anxiety, sleep quality, and quality of life were assessed using the Western Ontario and McMaster Universities Osteoarthritis Index (WOMAC), Lequesne Index (LI), Beck Depression Inventory (BDI), Beck Anxiety Inventory (BAI), Pittsburgh Sleep Quality Index (PSQI), and Short Form-36 (SF-36), respectively. **Results:** The frequency of NP was 46% according to the DN4 and 20% according to the PD-Q. Patients with NP had longer symptom duration, higher visual analog scale (VAS), WOMAC, LI, BAI, and PSQI scores, and lower SF-36 scores than patients without NP (all *p* < 0.05). NP scores correlated positively with VAS, WOMAC, LI, BDI, BAI, and PSQI scores and negatively with SF-36 scores. After adjustment for pain severity and symptom duration, several associations remained significant. **Conclusions:** NP was significantly associated with function, emotional status, sleep disturbance, and quality of life in patients with KOA.

## 1. Introduction

Knee osteoarthritis (KOA) is the most common chronic joint disorder and a major cause of disability worldwide. Pain, the main characteristic of symptomatic KOA, is associated with impaired functional status, an increased risk of disability, greater dependence on health care systems, and poor quality of life [1]. Knee pain and symptomatic KOA are highly prevalent among people aged 50 years and older worldwide, with a higher prevalence in women than in men [2]. A prospective, community-based study involving middle-aged women demonstrated a significant association between knee pain, with or without radiographic osteoarthritis (OA), and an increased risk of all-cause and cardiovascular disease-specific mortality [3]. Given the lack of effective treatments for OA and OA-related pain, and together with increasing life expectancy, OA has become an important public health concern at both the individual and societal levels [4].

Although KOA has traditionally been considered a model of inflammatory or nociceptive pain, patients with KOA may exhibit different pain phenotypes [5]. Structural changes have traditionally been assumed to generate nociceptive stimuli and have therefore been the primary targets of pain treatment. However, recent studies have demonstrated inconsistent associations between structural damage and pain [6]. In recent years, a growing body of evidence has supported the important role of central mechanisms and sensitization in the pathogenesis of OA [7]. Interactions between peripheral and central systems suggest plasticity within the nociceptive system in OA-related pain [8]. Heterogeneity in pain mechanisms among patients with OA may contribute to variable or poor responses to specific treatments [9].

Despite considerable variability in inclusion criteria, heterogeneity of the study populations, and differences in questionnaires used to assess neuropathic pain (NP), a growing number of studies suggest that NP mechanisms contribute to the pain experienced by at least a subset of patients with OA [10,11]. The present study aimed to determine the frequency of NP in patients with symptomatic KOA using the painDETECT questionnaire (PD-Q) and Douleur Neuropathique en 4 Questions (DN4) and to assess the association of NP with functional capacity, disease severity, emotional status, sleep disturbance, and quality of life.

## 2. Patients and Methods

This cross-sectional study included consecutive patients with painful KOA who presented to the outpatient clinic of physical medicine and rehabilitation between September 2019 and July 2020. All patients met the American College of Rheumatology classification criteria for KOA [12]. Patients with a history of cancer, systemic inflammatory disease, diabetes mellitus, psychiatric disorders, fibromyalgia, neurological conditions affecting sensory or motor function, or lower limb surgery were excluded from the study. Additional exclusion criteria included clinical findings suggestive of lumbar radiculopathy or lumbar spinal stenosis, and the use of medications specifically indicated for NP or antipsychotic medications. The study was approved by the local ethics committee of our institution and conducted in accordance with the principles of the Declaration of Helsinki. Written informed consent was obtained from all participants before enrollment.

### 2.1. Clinical Evaluation

Demographic and clinical data were collected for all patients. Knee pain severity was assessed using a visual analog scale (VAS) during activity (VASact), at night (VASn), and at rest (VASr). Anteroposterior radiographs of the knees were obtained, and radiographic severity was graded according to the Kellgren–Lawrence (KL) classification system, ranging from grade 0 (normal) to grade 4 (severe radiographic disease) [13]. Body weight and height were measured, and body mass index (BMI) was determined by dividing weight (kg) by height squared (m^2^).

All patients were evaluated using the Western Ontario and McMaster Universities Osteoarthritis Index (WOMAC) [14], the Lequesne Index (LI) [15], the Beck Depression Inventory (BDI) [16], the Beck Anxiety Inventory (BAI) [17], the Pittsburgh Sleep Quality Index (PSQI) [18], and Short Form-36 (SF-36) [19]. NP was assessed using the PD-Q [20] and the DN4 scale [21]. In the present study, the term “neuropathic pain (NP)” refers to neuropathic-like pain features identified using the DN4 and PD-Q screening questionnaires rather than to a definitive diagnosis of NP.

All patients included in the study had bilateral knee pain, and no single index knee was defined. Range of motion (ROM), crepitation, and radiographic KL grade were assessed separately for each knee, whereas VAS, WOMAC, and LI scores reflected the patients’ overall knee pain and symptoms rather than a specific knee. Clinical and radiographic assessments were performed by the same physician.

#### 2.1.1. Western Ontario and McMaster Universities Osteoarthritis Index

The WOMAC is a self-administered instrument specifically designed for individuals with hip or knee OA. It consists of 24 items divided into three subscales: pain, stiffness, and physical function. Higher scores indicate greater disease severity. The Turkish version of the WOMAC was shown to be valid and reliable by Tüzün et al. in 2005 [14].

#### 2.1.2. Lequesne Knee OA Index

The LI was used to determine the severity of KOA based on three clinical domains: pain, walking capacity, and activities of daily living. Increasing LI scores correspond to greater disease severity. The Turkish adaptation of the LI was previously shown to have adequate validity and reliability by Basaran et al. [15].

#### 2.1.3. Beck Depression Inventory

Depressive symptoms were evaluated using the BDI, a self-report instrument comprising 21 items. The total score reflects the severity of depressive symptoms, with higher values indicating greater severity. Scores are classified into four categories: minimal (0–9), mild (10–18), moderate (19–29), and severe depression (30–63). The Turkish adaptation of the BDI, previously validated by Hisli et al., was used in this study [16].

#### 2.1.4. Beck Anxiety Inventory

The BAI is a brief, 21-item self-report questionnaire used to assess the severity of anxiety symptoms. The total score ranges from 0 to 63, with higher scores indicating greater anxiety severity. According to the BAI scores, anxiety severity was categorized as normal or no anxiety (0–9), mild-to-moderate anxiety (10–18), moderate-to-severe anxiety (19–29), and severe anxiety (30–63). The Turkish version of the BAI was validated by Ulusoy et al. [17].

#### 2.1.5. Pittsburgh Sleep Quality Index

The PSQI is a 19-item questionnaire used to assess sleep quality and sleep-related disturbances during the previous month. It comprises seven domains: subjective sleep quality, sleep latency, sleep duration, habitual sleep efficiency, sleep disturbances, use of sleep medication, and daytime dysfunction. Patients with a total PSQI score ≥ 5 were classified as having poor sleep quality. The Turkish version of the PSQI was validated by Ağargün et al. [18].

#### 2.1.6. Short Form-36

The SF-36 is a widely used questionnaire for assessing quality of life across eight domains of physical and emotional health. It evaluates physical functioning (PF), role limitations due to physical health problems (RP), bodily pain (P), general health perceptions (GH), vitality (VT), social functioning (SF), role limitations due to emotional problems (RE), and mental health (MH). The Turkish version of the SF-36 was validated by Kocyiğit et al. [19].

#### 2.1.7. PainDETECT Questionnaire

The PD-Q is a nine-item patient-reported measure used to assess pain intensity, quality, pattern, and radiation. Total scores range from 0 to 38, with higher values reflecting more prominent neuropathic-like symptoms. For dichotomous comparisons, patients with PD-Q scores ≥ 19 were classified as having NP, whereas those with scores < 19, including the uncertain range of 13–18, were included in the no-NP group. The Turkish version of the PD-Q was validated by Alkan et al. in 2013 [20].

#### 2.1.8. Douleur Neuropathique en 4 Questions (DN4)

The DN4 is a clinician-administered questionnaire containing 10 items with yes/no responses. Positive responses receive 1 point, whereas negative responses receive 0 points, yielding a total score between 0 and 10. Patients with a DN4 score ≥ 4 were classified as having NP. The Turkish version of the DN4 was validated by Celik et al. [21].

### 2.2. Statistical Analysis

Statistical analyses were carried out using SPSS for Windows, version 18.0 (SPSS, Chicago, IL, USA). Categorical data were presented as numbers and percentages, whereas continuous data were reported as mean ± standard deviation or median (range), as appropriate. The normality of the data distribution was assessed using the Kolmogorov–Smirnov test. Comparisons between groups were performed with Student’s t-test for normally distributed variables and the Mann–Whitney U test for variables with a non-normal distribution. Categorical variables were compared using the chi-square test or Fisher’s exact test, as appropriate. Relationships between variables were examined using Pearson’s or Spearman’s correlation analysis according to the data distribution. Agreement between the DN4 and PD-Q in identifying NP was assessed using Cohen’s kappa coefficient (κ). Effect sizes were reported according to the statistical test used. Hedges’ g was calculated for parametric continuous variables, with approximate 95% confidence intervals based on its standard error. Rank-biserial correlation (r_rb) was used for Mann–Whitney U comparisons, with 95% confidence intervals obtained by bootstrap resampling with 3000 iterations. Hodges–Lehmann estimates and their 95% confidence intervals were also obtained using bootstrap resampling. Odds ratios (ORs) were used for binary categorical variables, with 95% confidence intervals calculated on the logarithmic scale. Cramér’s V was used for categorical variables with more than two levels, with 95% confidence intervals obtained using 3000 bootstrap resamples. Limited multivariable linear regression analyses were performed with adjustment for pain severity (VAS activity) and symptom duration. Regression assumptions were evaluated using residual diagnostics, and robust standard errors were used. Outcomes showing marked floor or ceiling effects and poor fit with linear regression assumptions were excluded from the adjusted analyses. Statistical significance was defined as a *p*-value < 0.05.

## 3. Results

The demographic and clinical characteristics of the patients with KOA are presented in Table 1. The mean age of patients was 58.8 ± 7.2 years. Most patients were female (79%) and obese, with a mean BMI of 32.3 ± 5.0. The median symptom duration was 36 months. The mean VASact score was 6.1 ± 1.8, whereas the median pain VASr and VASn scores were 1 (range, 0–6), and 2 (range, 0–10), respectively. The frequency of NP among patients with KOA was 46% according to the DN4 and 20% according to the PD-Q. Cross-classification of the two screening instruments showed that 20 patients were classified as positive by both DN4 and PD-Q. All PD-Q-positive patients were also DN4-positive, whereas 26 additional patients were positive only according to DN4. The agreement between the DN4 and PD-Q was moderate (Cohen’s κ = 0.454, *p* < 0.001).

Based on the DN4 scale, comparisons between KOA patients with NP (*n* = 46) and those without NP (*n* = 54) are presented in Table 2. No significant differences were observed between the groups in terms of age, gender, BMI, presence of crepitation, left knee range of motion (ROM), or KL grade (all *p* > 0.05). In contrast, patients with NP had significantly longer symptom duration and duration of morning stiffness, as well as higher pain scores (VASact, VASr, and VASn), than those without NP (60 vs. 24 months, 10 vs. 2 min, 6.9 ± 1.5 vs. 5.3 ± 1.7, 2 vs. 0, and 4 vs. 1, respectively; all *p* < 0.001). The presence of morning stiffness was also significantly higher in the NP group (95.7% vs. 81.5%, *p* = 0.030). Right knee ROM was also significantly lower in the NP group (125 ± 10 vs. 129 ± 7 degrees, *p* = 0.033). Furthermore, patients with NP had significantly higher PD-Q, WOMAC, LI, BAI, and PSQI scores (17.6 ± 5.7 vs. 5.5 ± 3.1, *p* < 0.001; 44 vs. 21.5, *p* < 0.001; 12.4 ± 3.3 vs. 8.5 ± 3.5, *p* < 0.001; 10 vs. 6.5, *p* = 0.005; 6.4 ± 2.7 vs. 5.0 ± 2.5, *p* = 0.010, respectively), and lower SF-36 scores in the PF, SF, and P subscales (48.4 ±17.4 vs. 62.5 ± 17.4, *p* < 0.001; 46.2 ± 20.3 vs. 64.2 ± 21.1, *p* = 0.001; 29.3 ± 18.4 vs. 49.1 ± 17.9, *p* < 0.001, respectively). No significant difference was observed in BDI scores between the groups (*p* > 0.05).

Based on the PD-Q, comparisons between KOA patients with NP (*n* = 20) and those without NP (*n* = 80) are presented in Table 3. No significant differences were observed between the groups in terms of age, gender, BMI, presence of knee crepitation, left knee ROM, or KL grade (all *p* > 0.05). However, patients with NP had a significantly longer symptom duration and duration of morning stiffness and significantly higher VASact, VASr, and VASn pain scores than those without NP (60 vs. 36 months; 15 vs. 5 min; 7.4 ± 1.1 vs. 5.8 ± 1.7; 2.5 vs. 0; 5.5 vs. 1, respectively; all *p* < 0.05). Right knee ROM was also significantly lower in the NP group (123 ± 12 vs. 128 ± 7 degrees, *p* = 0.043). Patients with NP demonstrated significantly higher scores in DN4, WOMAC, LI, BAI, BDI, and PSQI compared to those without NP (all *p* ≤ 0.002). Regarding health-related quality of life, patients with NP had significantly lower SF-36 PF, SF, and P scores than patients without NP (all *p* ≤ 0.002).

The associations of DN4 and PD-Q scores with disease-related parameters are presented in Table 4. Both PD-Q and DN4 scores were positively correlated with symptom duration, duration of morning stiffness, pain scores (VASact, VASr, and VASn), and WOMAC, LI, BDI, BAI, and PSQI scores (all *p* < 0.05). In contrast, both scores showed negative correlations with right knee ROM and SF-36 subscales including PF, SF, and P (all *p* < 0.05). KL grade showed a weak positive correlation with DN4 scores but no significant correlation with PD-Q scores (rho = 0.280, *p* = 0.005; rho = 0.180, *p* = 0.073, respectively). Additionally, PD-Q scores were negatively correlated with the RP and RE subscales of the SF-36 (both *p* < 0.05). A strong positive correlation was observed between PD-Q and DN4 scores (r = 0.897, *p* < 0.001).

In limited adjusted analyses controlling for pain severity (VAS activity) and symptom duration, DN4-defined NP remained significantly associated with higher BAI (adjusted B = 3.63, 95% CI 1.02 to 6.25; *p* = 0.006), PSQI (B = 1.35, 95% CI 0.08 to 2.62; *p* = 0.038), and WOMAC scores (B = 10.01, 95% CI 4.00 to 16.02; *p* = 0.001), as well as lower SF-36 pain scores (B = −9.53, 95% CI −17.47 to −1.59; *p* = 0.019). The association with BDI was not statistically significant after adjustment (B = 1.15, 95% CI −1.51 to 3.82; *p* = 0.392). Similarly, PD-Q-defined neuropathic-like pain remained significantly associated with higher BDI (B = 5.49, 95% CI 2.51 to 8.47; *p* < 0.001), BAI (B = 3.91, 95% CI 1.15 to 6.68; *p* = 0.005), PSQI (B = 2.35, 95% CI 1.01 to 3.69; *p* < 0.001), and WOMAC scores (B = 14.46, 95% CI 6.66 to 22.27; *p* < 0.001), and with lower SF-36 social function (B = −12.40, 95% CI −23.81 to −0.99; *p* = 0.033) and pain scores (B = −13.53, 95% CI −23.70 to −3.35; *p* = 0.009). The SF-36 RP domain was excluded from the adjusted analyses because of a marked floor effect and poor fit with linear regression assumptions. Other SF-36 domains were not significantly associated with NP group status after adjustment (Table 5).

## 4. Discussion

The present study demonstrated that KOA patients with NP had longer symptom duration, higher pain scores, and greater impairments in physical function, emotional status, sleep quality, and quality of life. Moreover, NP scores were significantly associated with longer symptom duration, greater pain intensity and functional impairment, reduced knee ROM, increased depressive and anxiety symptoms, poorer sleep quality and reduced quality of life.

Osteoarthritis is the most common musculoskeletal disorder and is often accompanied by functional limitations and reduced quality of life [10]. Chronic pain is the most important symptom of OA. Both structural joint changes and abnormal excitability in peripheral and central pain pathways contribute to the pathophysiology of chronic pain. Growing evidence suggests that NP may contribute, at least in part, to pain in OA. A systematic review estimated the prevalence of NP in knee or hip OA to be approximately 23% [11]. Another recent systematic review and meta-analysis reported that the prevalence of NP and/or pain sensitization in the KOA ranged from 20% to 40%, regardless of the questionnaire used or the population studied [22]. Among the available assessment tools, the PD-Q was the most frequently used across various clinical settings.

In the present study, in addition to the PD-Q, the DN4 scale was used to evaluate neuropathic-like pain features in KOA. The frequency of NP was 46% based on the DN4 scale and 20% based on the PD-Q. Only PD-Q scores ≥19 were considered indicative of likely NP features in our study. This threshold may have contributed to the lower frequency observed with the PD-Q. Beyond this, differences in item structure between the two instruments may also have contributed to the discrepancy in prevalence estimates. Despite the strong positive correlation between DN4 and PD-Q scores (r = 0.897), agreement between the two instruments in identifying NP was only moderate (κ = 0.454). Notably, all patients classified as positive by the PD-Q were also positive according to the DN4, whereas 26 additional patients were classified as positive only by the DN4. These findings suggest that the two instruments assess related but not identical neuropathic pain features and may therefore identify partially overlapping patient groups. Accordingly, the DN4 and PD-Q may not be fully interchangeable when used to identify NP in patients with KOA.

Variable prevalence estimates have also been reported in the literature. Yıldırım et al. observed that 40% of patients with KOA had NP according to the DN4 scale, whereas Moss et al. reported a frequency of 22.3% using the PD-Q [5,23]. Aşkın et al. also used both tools and reported NP frequencies of 66.7% with the PD-Q and 46.7% with the DN4 [24]. Conversely, other studies have reported lower frequencies of NP components in KOA, ranging from 5% to 11% [25,26,27]. These variations may reflect differences in study populations as well as in the characteristics and thresholds of the screening instruments used.

In the present study, we also evaluated the association between NP and disease-related clinical features and demonstrated that KOA patients with NP had longer symptom duration and duration of morning stiffness, as well as higher pain (VASact, VASn, and VASr), WOMAC, and LI scores, compared with patients without NP, based on both the PD-Q and DN4. Notably, despite these differences in clinical symptoms and functional impairment, radiographic severity did not differ significantly between patients with and without NP according to either the DN4 or PD-Q. Moreover, both NP scores were positively correlated with symptom duration, duration of morning stiffness, pain, WOMAC, and LI scores, and negatively correlated with right knee ROM. Similar to our findings, Aşkın et al. reported that both PD-Q and DN4 scores were significantly associated with WOMAC scores in patients with KOA [24]. In that study, VASr scores were positively correlated with PD-Q scores but not with DN4 scores. Yıldırım et al. demonstrated that pain (VASr and VASact) and LI scores were higher in KOA patients with NP than those without NP and that DN4 scores were correlated with LI scores [23]. In contrast to our findings, Ohtori et al. found no significant association between PD-Q scores and symptom duration or KL grade in patients with KOA [25]. Consistent with our findings, they also reported positive correlations of PD-Q scores with WOMAC and VAS scores. Oteo-Alvaro et al. demonstrated that patients with KOA who had NP based on the DN4 scale had more severe pain, longer symptom duration, higher KL grade, and greater structural damage than patients without NP [28]. In contrast, van Helvoort et al. reported that patients with NP had significantly less radiographic damage in their index knee but significantly greater impairment in physical function than patients without NP [26]. However, in the present study, KL grade showed a weak but significant positive correlation with DN4 scores but not with PD-Q scores. The discordance between neuropathic-like pain and radiographic severity in KOA has also been reported in previous studies and has been attributed to central sensitization [29,30].

Neuropathic pain is frequently associated with comorbid sleep disorders, anxiety, and depression, which can substantially impair quality of life, adversely affect treatment outcomes, and increase morbidity and mortality [31]. The relationship between NP and these comorbidities is complex, and the underlying mechanisms remain unclear. Pain may exacerbate comorbid conditions, which, in turn, may contribute to greater pain severity. Major depressive disorder has been determined to be associated with alterations in signaling pathways involved in neuroplasticity and cell survival [32]. Experimental evidence also suggests that chronic pain may induce depressive-like behavior through neuroplastic changes in brain regions involved in the regulation of both pain and emotion [33]. In the present study, KOA patients with NP, as identified by either the PD-Q or DN4, had significantly higher BAI scores and lower SF-36 subdomain scores for PF, SF, and P than those without NP. Although BDI scores did not differ significantly between patients with and without NP according to the DN4, they were significantly higher in patients with NP identified by the PD-Q. Additionally, both DN4 and PD-Q scores demonstrated a statistically significant positive correlation with BAI and BDI scores and a negative correlation with SF-36 scores. Importantly, the associations of NP status with anxiety, sleep quality, and the SF-36 pain domain remained significant for both instruments after adjustment for pain severity and symptom duration. The association with depressive symptoms persisted only for the PD-Q classification. These findings suggest that the relationships between NP and psychological or sleep-related outcomes may not be fully attributable to pain intensity or symptom duration, although residual confounding cannot be excluded. Aşkın et al. found no significant differences in Hospital Anxiety and Depression (HAD) scores between KOA patients with and without NP according to either the DN4 or PD-Q; however, DN4 scores were negatively correlated with SF-36 scores [24]. Mesci et al. reported higher HAD scores and lower SF-36 scores in KOA patients with NP identified by the PD-Q than in those without NP [34]. Similarly, Hochman et al. demonstrated that higher levels of both depressive symptoms and pain catastrophizing were associated with the presence of NP symptoms using a modified PD-Q in a community-based KOA cohort [35]. In contrast, Yıldırım et al. found no significant differences in SF-36 or BDI scores between KOA patients with and without NP according to the DN4 [23]. In addition, sleep quality was poorer in KOA patients with NP in the present study, and PSQI scores were significantly associated with both PD-Q and DN4 scores. Evidence regarding the relationship between NP and sleep quality in patients with KOA remains limited. Illeez et al. reported significant improvements in PSQI scores following pregabalin and duloxetine treatment in KOA patients with NP compared with pretreatment values [36]. Previous studies have also demonstrated poor sleep quality among patients with NP across different underlying causes of neuropathic pain [37,38].

This study has several limitations. First, its cross-sectional and single-center design precludes causal inference and may limit the generalizability of the findings. Second, although limited multivariable analyses were performed adjusting for pain severity and symptom duration, residual confounding by unmeasured or unrecorded factors cannot be excluded. Third, the DN4 and PD-Q are screening tools for neuropathic-like pain features rather than diagnostic instruments. In addition, patients with PD-Q scores of 13–18 were included in the PD-Q-negative group, which may have introduced some misclassification. The relatively small sample size, particularly the number of PD-Q-positive patients, together with the absence of an a priori sample size calculation, may have limited statistical power and reduced the stability and precision of the corresponding multivariable estimates. Accordingly, the adjusted PD-Q analyses should be interpreted cautiously. Moreover, the large number of statistical comparisons without adjustment for multiple testing may have increased the risk of type I error. The lack of formal blinding of the radiographic assessment to clinical findings and the absence of intra-observer reliability testing may have introduced observer-related measurement bias. The absence of an asymptomatic control group and the lack of systematic control for analgesic use should also be considered. Finally, the extensive exclusion criteria resulted in a selected population, which may limit the generalizability of the findings to the broader KOA population.

The strengths of the study include the evaluation of the NP component using two different questionnaires and the assessment of its association with several disease-related characteristics, such as pain, function, emotional status, sleep disturbance, and quality of life.

## 5. Conclusions

Neuropathic pain features were common among patients with KOA, although their prevalence varied according to the screening instrument used. Higher DN4 and PD-Q scores were associated with greater pain severity and functional impairment, as well as poorer sleep and selected psychological and quality-of-life outcomes. Given the cross-sectional design of the study, these findings do not establish causal relationships. Prospective studies are needed to clarify the clinical significance of NP and their potential implications for the management of KOA.

## Figures and Tables

**Table 1:** Demographic and clinical characteristics of patients with knee osteoarthritis.

	Patients with KOA (*n* = 100)
Age (years)	58.8 ± 7.2
Female/male *n* (%)	79/21 (79/21)
BMI (kg/m^2^)	32.3 ± 5.0
Symptom duration, months	36 (3–300)
Presence of morning stiffness, *n* (%)	88 (88)
Duration of morning stiffness, minute	5 (1–30)
Presence of right knee crepitation, *n* (%)	69 (69)
Presence of left knee crepitation, *n* (%)	72 (72)
Right knee ROM, degree	127 ± 8
Left knee ROM, degree	127 ± 7
KL grade, *n* (%)	
Grade I	13 (13)
Grade II	50 (50)
Grade III	26 (26)
Grade IV	11 (11)
VAS activity	6.1 ± 1.8
VAS rest	1 (0–6)
VAS night	2 (0–10)
WOMAC	30.5 (3–84)
DN4	3.3 ± 2.2
PD-Q	11.0 ± 7.5
Lequesne Index	10.3 ± 3.9
BDI	13.5 (1–31)
BAI	10 (0–30)
PSQI	5.7 ± 2.7
Short Form-36	
Physical function	56.1 ± 18.7
Role-physical	0 (0–100)
Role emotional	83.3 (0–100)
Vitality	52.6 ± 17.1
Mental health	65.2 ± 13.7
Social function	60.6 ± 22.0
Pain	45.2 ± 16.5
General health	43.2 ± 16.5

Data are presented as mean ± standard deviation or median (min–max), as appropriate. ROM, range of motion; KL, Kellgren–Lawrence; VAS, visual analog scale; WOMAC, Western Ontario and McMaster Universities Osteoarthritis Index; DN4, Douleur Neuropathique en 4 Questions; PD-Q, painDETECT Questionnaire; BMI, body mass index; BDI, Beck Depression Inventory; BAI, Beck Anxiety Inventory; PSQI, Pittsburgh Sleep Quality Index.

**Table 2:** Comparison of knee osteoarthritis patients with and without neuropathic pain according to the DN4 scale.

	DN4 ≥ 4 (*n* = 46)	DN4 < 4 (*n* = 54)	Effect Size (95% CI)	*p* Value
Age (years)	58.0 ± 8.1	59.5 ± 6.3	−0.22 (−0.61 to 0.18)	0.274 ^†^
Female/male *n* (%)	36/10 (78.3/21.7)	43/11 (79.6/20.4)	0.92 (0.35 to 2.42)	0.867 ^††^
BMI (kg/m^2^)	33.4 ± 5.0	31.5 ± 5.0	0.36 (−0.03 to 0.76)	0.070 ^†^
Symptom duration (months)	54(12–240)	24 (3–300)	0.43 (0.22 to 0.62)	<0.001 ^††^
Presence of morning stiffness, *n* (%)	44 (95.7)	44 (81.5)	5.00 (1.04 to 24.15)	0.030 ^†††^
Duration of morning stiffness (minute)	10 (2–30)	3.5 (1–25)	0.51 (0.31 to 0.69)	<0.001 ^††^
Presence of right knee crepitation, *n* (%)	35(76.1)	34 (63.0)	1.87 (0.78 to 4.49)	0.157 ^††††^
Presence of left knee crepitation, *n* (%)	36 (78.3)	36 (66.7)	1.80 (0.73 to 4.43)	0.198 ^††††^
Right knee ROM	125 ± 10	129 ± 7	−0.44 (−0.84 to −0.05)	0.033 ^†^
Left knee ROM	126 ± 8	128 ± 7	−0.22 (−0.62 to 0.17)	0.270 ^†^
KL grade *n* (%)			0.25 (0.12 to 0.44)	0.108 ^†††^
Grade I	3 (6.5)	10 (18.5)		
Grade II	22 (47.8)	28 (51.9)		
Grade III	13 (28.3)	13 (24)		
Grade IV	8 (17.4)	3 (5.6)		
VAS activity	6.9 ± 1.5	5.3 ± 1.7	0.97 (0.55 to 1.38)	<0.001 ^†^
VAS rest	2 (0–6)	0 (0–5)	0.53 (0.35 to 0.71)	<0.001 ^††^
VAS night	4 (0–10)	1 (0–8)	0.66 (0.49 to 0.82)	<0.001 ^††^
WOMAC	44 (16–84)	21.5 (3–80)	0.59 (0.42 to 0.75)	<0.001 ^††^
Lequesne Index	12.4 ± 3.3	8.5 ± 3.5	1.12 (0.70 to 1.54)	<0.001 ^†^
PD-Q	17.6 ± 5.7	5.5 ± 3.1	2.65 (2.11 to 3.19)	<0.001 ^†^
BDI	13.5 (1–31)	8 (0–26)	0.20 (−0.04 to 0.42)	0.086 ^††^
BAI	10 (0–30)	6.5 (1–21)	0.32 (0.10 to 0.52)	0.005 ^††^
PSQI	6.4 ± 2.7	5.0 ± 2.5	0.52 (0.12 to 0.92)	0.010 ^†^
Short Form-36				
Physical function	48.4 ± 17.4	62.5 ± 17.4	−0.80 (−1.21 to −0.39)	<0.001 ^†^
Role—physical	0 (0–100)	25 (0–100)	−0.18 (−0.38 to 0.02)	0.087 ^††^
Role—emotional	66.7 (0–100)	100 (0–100)	−0.15 (−0.37 to 0.07)	0.164 ^††^
Vitality	52.5 ± 17.0	52.6 ± 17.4	−0.01 (−0.40 to 0.38)	0.958 ^†^
Mental health	65.3 ± 13.7	65.1 ± 13.8	0.01 (−0.38 to 0.40)	0.966 ^†^
Social function	54.8 ± 21.3	65.5 ± 21.7	−0.49 (−0.89 to −0.09)	0.016 ^†^
Pain	36.5 ± 18.5	52.5 ± 17.5	−0.88 (−1.30 to −0.47)	<0.001 ^†^
General health	41.1 ± 17.8	45.0 ± 15.2	−0.23 (−0.62 to 0.17)	0.253 ^†^

Data are presented as mean ± standard deviation or median (min–max), as appropriate. Effect sizes are presented as Hedges’ g for parametric continuous variables, rank-biserial correlation (r_rb) for Mann–Whitney U comparisons, odds ratios (ORs) for binary categorical variables, and Cramér’s V for categorical variables with more than two levels. For r_rb and Cramér’s V, 95% confidence intervals were obtained using 3000 bootstrap resamples; 95% confidence intervals for ORs were calculated on the logarithmic scale. ^†^ Student’s *t*-test; ^††^ Mann–Whitney U test; ^†††^ Fisher exact test; ^††††^ chi-square test. DN4, Douleur Neuropathique en 4 Questions; BMI, body mass index; ROM, range of motion; VAS, visual analog scale; WOMAC, Western Ontario and McMaster Universities Osteoarthritis Index; BDI, Beck Depression Inventory; BAI, Beck Anxiety Inventory; PSQI, Pittsburgh Sleep Quality Index.

**Table 3:** Comparison of knee osteoarthritis patients with and without neuropathic pain according to the PD-Q.

	PD-Q ≥ 19 (*n* = 20)	PD-Q < 19 (*n* = 80)	Effect Size (95% CI)	*p* Value
Age (years)	55.2 ± 9.3	59.8 ± 6.4	−0.64 (−1.13 to −0.14)	0.051 ^†^
Female/male *n* (%)	15/5 (75/25)	64/16 (80/20)	0.75 (0.24 to 2.37)	0.623 ^††^
BMI (kg/m^2^)	34.2 ± 5.8	31.9 ± 4.7	0.45 (−0.04 to 0.95)	0.071 ^†^
Symptom duration (months)	60 (12–120)	36 (3–300)	0.38 (0.14 to 0.61)	0.008 ^††^
Presence of morning stiffness, *n* (%)	20 (100)	68 (85)	7.48 (0.42 to 131.89)	0.065 ^†††^
Duration of morning stiffness (minute)	12.5 (5–25)	5 (1–30)	0.58 (0.38 to 0.75)	<0.001 ^††^
Presence of right knee crepitation, *n* (%)	13 (65)	56 (70)	0.80 (0.28 to 2.24)	0.665 ^††††^
Presence of left knee crepitation, *n* (%)	17 (85)	55 (68.8)	2.58 (0.69 to 9.60)	0.148 ^†††^
Right knee ROM	123 ± 12	128 ± 7	−0.51 (−1.00 to −0.01)	0.043 ^†^
Left knee ROM	127 ± 7	127 ± 8	−0.09 (−0.58 to 0.40)	0.730 ^†^
KL grade, *n* (%)			0.09 (0.06 to 0.35)	0.833 ^†††^
Grade I	2 (10)	11 (13.8)		
Grade II	9 (45)	41 (51.3)		
Grade III	6 (30)	20 (25)		
Grade IV	3 (15)	8 (10)		
VAS activity	7.4 ± 1.1	5.8 ± 1.7	0.94 (0.43 to 1.44)	<0.001 ^†^
VAS rest	2.5 (0–6)	0 (0–5)	0.67 (0.47 to 0.83)	<0.001 ^††^
VAS night	5.5 (2–9)	1 (0–10)	0.82 (0.69 to 0.92)	<0.001 ^††^
WOMAC	54.5 (25–74)	24.5 (3–84)	0.70 (0.52 to 0.86)	<0.001 ^††^
Lequesne Index	14.2 ± 2.2	9.4 ± 3.6	1.39 (0.87 to 1.92)	<0.001 ^†^
DN4	6.2 ± 1.1	2.6 ± 1.8	2.01 (1.45 to 2.58)	<0.001 ^†^
BDI	16 (1–31)	8 (0–26)	0.50 (0.22 to 0.74)	0.001 ^††^
BAI	11.5 (4–20)	7 (0–30)	0.45 (0.23 to 0.66)	0.002 ^††^
PSQI	7.6 ± 2.2	5.2 ± 2.6	0.90 (0.40 to 1.41)	<0.001 ^†^
Short Form-36				
Physical function	46.7 ± 12.3	58.4 ± 19.4	−0.63 (−1.13 to −0.14)	0.002 ^†^
Role—physical	0 (0–100)	0 (0–100)	−0.18 (−0.41 to 0.07)	0.167 ^††^
Role—emotional	66.7 (0–100)	100 (0–100)	−0.15 (−0.42 to 0.14)	0.279 ^††^
Vitality	48.7 ± 18.9	53.5 ± 16.7	−0.28 (−0.77 to 0.21)	0.265 ^†^
Mental health	62.8 ± 13.9	65.8 ± 13.6	−0.22 (−0.71 to 0.27)	0.377 ^†^
Social function	46.2 ± 20.3	64.2 ± 21.1	−0.85 (−1.35 to −0.35)	0.001 ^†^
Pain	29.3 ± 18.4	49.1 ± 17.9	−1.09 (−1.60 to −0.58)	<0.001 ^†^
General health	40.5 ± 20.3	43.9 ± 15.5	−0.21 (−0.70 to 0.28)	0.408 ^†^

Data are presented as mean ± standard deviation or median (min–max), as appropriate. Effect sizes are presented as Hedges’ g for parametric continuous variables, rank-biserial correlation (r_rb) for Mann–Whitney U comparisons, odds ratios (ORs) for binary categorical variables, and Cramér’s V for categorical variables with more than two levels. For r_rb and Cramér’s V, 95% confidence intervals were obtained using 3000 bootstrap resamples; 95% confidence intervals for ORs were calculated on the logarithmic scale. ^†^ Student’s *t* test; ^††^ Mann–Whitney U test; ^†††^ Fisher’s exact test; ^††††^ chi-square test. PD-Q, painDETECT Questionnaire; BMI, body mass index; ROM, range of motion; VAS, visual analog scale; WOMAC, Western Ontario and McMaster Universities Osteoarthritis Index; BDI, Beck Depression Inventory; BAI, Beck Anxiety Inventory; PSQI, Pittsburgh Sleep Quality Index.

**Table 4:** The association of DN4 and PD-Q scores with demographic and clinical characteristics.

	DN4		PD-Q	
	Correlation Coefficient	*p*-Value	Correlation Coefficient	*p*-Value
Age	−0.132 ^†^	0.189	−0.155 ^†^	0.124
BMI	0.228 ^†^	0.023	0.189 ^†^	0.060
Symptom duration	0.420 ^††^	<0.001	0.363 ^††^	<0.001
Morning stiffness duration	0.512 ^††^	<0.001	0.524 ^††^	<0.001
Right knee ROM	−0.294 ^†^	0.003	−0.290 ^†^	0.003
Left knee ROM	−0.182 ^†^	0.070	−0.145 ^†^	0.149
K-L grade	0.280 ^††^	0.005	0.180 ^††^	0.073
VAS activity	0.425 ^†^	<0.001	0.440 ^†^	<0.001
VAS rest	0.522 ^††^	<0.001	0.520 ^††^	<0.001
VAS night	0.682 ^††^	<0.001	0.706 ^††^	<0.001
WOMAC	0.586 ^††^	<0.001	0.593 ^††^	<0.001
Lequesne Index	0.579 ^†^	<0.001	0.575 ^†^	<0.001
BDI	0.256 ^††^	0.010	0.275 ^†^	0.006
BAI	0.329 ^††^	<0.001	0.380 ^††^	<0.001
PSQI	0.341 ^††^	<0.001	0.400 ^††^	<0.001
Short Form-36				
Physical function	−0.389 ^†^	<0.001	−0.377 ^†^	<0.001
Role physical	−0.193 ^††^	0.055	−0.197 ^††^	0.049
Role emotional	−0.164 ^††^	0.103	−0.221 ^††^	0.027
Vitality	−0.104 ^†^	0.302	−0.099 ^†^	0.329
Mental health	−0.058 ^†^	0.569	−0.033 ^†^	0.745
Social function	−0.256 ^†^	0.010	−0.274 ^†^	0.006
Pain	−0.421 ^†^	<0.001	−0.506 ^†^	<0.001
General health	−0.122 ^†^	0.225	−0.112 ^†^	0.267

^†^ r Pearson correlation coefficient; ^††^ rho Spearman correlation coefficient. PD-Q, painDETECT Questionnaire; DN4, Douleur Neuropathique en 4 Questions; BMI, body mass index; ROM, range of motion; VAS, visual analog scale; WOMAC, Western Ontario and McMaster Universities Osteoarthritis Index; BDI, Beck Depression Inventory; BAI, Beck Anxiety Inventory; PSQI, Pittsburgh Sleep Quality Index.

**Table 5:** Limited adjusted associations between neuropathic pain group status and selected patient-reported outcomes.

	DN4 ≥ 4		PD-Q ≥ 19	
	Adjusted B (95% CI)	*p*-Value	Adjusted B (95% CI)	*p*-Value
BDI	1.15 (−1.51 to3.82)	0.392	5.49 (2.51 to 8.47)	<0.001
BAI	3.63 (1.02 to 6.25)	0.006	3.91 (1.15 to 6.68)	0.005
PSQI	1.35 (0.08 to 2.62)	0.038	2.35 (1.01 to 3.69)	<0.001
WOMAC	10.01 (4.00 to 16.02)	0.001	14.46 (6.66 to 22.27)	<0.001
Short Form-36				
Physical function	−6.94 (−14.17 to 0.28)	0.060	−4.38 (−12.80 to 4.04)	0.308
Role emotional	−6.58 (−22.20 to 9.03)	0.409	−11.23 (−33.30 to 10.84)	0.319
Vitality	2.97 (−5.04 to 10.98)	0.468	−3.20 (−12.96 to 6.55)	0.520
Mental health	2.23 (−4.36 to 8.82)	0.507	−2.02 (−9.54 to 5.51)	0.600
Social function	−4.08 (−13.35 to 5.20)	0.389	−12.40 (−23.81 to −0.99)	0.033
Pain	−9.53 (−17.47 to −1.59)	0.019	−13.53 (−23.70 to −3.35)	0.009
General health	−1.81 (−9.57 to 5.96)	0.648	−1.51 (−11.80 to 8.77)	0.773

Values are unstandardized regression coefficients (B) with 95% confidence intervals (CIs). Separate multivariable linear regression models were fitted for DN4 group status and PD-Q group status. All models were adjusted for pain severity (VAS activity) and symptom duration (months). BDI, Beck Depression Inventory; BAI, Beck Anxiety Inventory; PSQI, Pittsburgh Sleep Quality Index; WOMAC, Western Ontario and McMaster Universities Osteoarthritis Index; SF-36, 36-Item Short Form Health Survey; VAS, visual analog scale; CI, confidence interval.

## Data Availability

The data presented in this study are available upon reasonable request from the corresponding author. The data are not publicly available due to privacy and ethical restrictions.

## References

[1] Moore R.L., Clifford A.M., Moloney N., Doody C., Smart K.M., O’Leary H. (2020). The relationship between clinical and quantitative measures of pain sensitization in knee osteoarthritis. Clin. J. Pain.

[2] Busija L., Bridgett L., Williams S.R.M., Osborne R.H., Buchbinder R., March L., Fransen M. (2010). Osteoarthritis. Best Pract. Res. Clin. Rheumatol..

[3] Kluzek S., Sanchez-Santos M.T., Leyland K.M., Judge A., Spector T.D., Hart D., Cooper C., Newton J., Arden N.K. (2016). Painful knee but not hand osteoarthritis is an independent predictor of mortality over 23 years follow-up of a population-based cohort of middle-aged women. Ann. Rheum. Dis..

[4] Neogi T. (2013). The epidemiology and impact of pain in osteoarthritis. Osteoarthr. Cartil..

[5] Moss P., Benson H.A.E., Will R., Wright A. (2018). Patients with knee osteoarthritis who score highly on the PainDETECT questionnaire present with multimodality hyperalgesia, increased pain, and impaired physical function. Clin. J. Pain.

[6] Previtali D., Capone G., Marchettini P., Candrian C., Zaffagnini S., Filardo G. (2022). High prevalence of pain sensitization in knee osteoarthritis: A meta-analysis with meta-regression. Cartilage.

[7] Trouvin A.P., Perrot S. (2018). Pain in osteoarthritis. Implications for optimal management. Jt. Bone Spine.

[8] Perrot S. (2015). Osteoarthritis pain. Best Pract. Res. Clin. Rheumatol..

[9] Moreton B.J., Tew V., das Nair R., Wheeler M., Walsh D.A., Lincoln N.B. (2015). Pain phenotype in patients with knee osteoarthritis: Classification and measurement properties of painDETECT and self-report Leeds Assessment of Neuropathic Symptoms and Signs scale in a cross-sectional study. Arthritis Care Res..

[10] Dimitroulas T., Duarte R.V., Behura A., Kitas G.D., Raphael J.H. (2014). Neuropathic pain in osteoarthritis: A review of pathophysiological mechanisms and implications for treatment. Semin. Arthritis Rheum..

[11] French H.P., Smart K.M., Doyle F. (2017). Prevalence of neuropathic pain in knee or hip osteoarthritis: A systematic review and meta-analysis. Semin. Arthritis Rheum..

[12] Altman R., Asch E., Bloch D., Bole G., Borenstein D., Brandt K., Christy W., Cooke T.D., Greenwald R., Hochberg M. (1986). Development of criteria for the classification and reporting of osteoarthritis. Classification of osteoarthritis of the knee. Diagnostic and Therapeutic Criteria Committee of the American Rheumatism Association. Arthritis Rheum..

[13] Kellgren J.H., Lawrence J.S. (1957). Radiological assessment of osteo-arthrosis. Ann. Rheum. Dis..

[14] Tüzün E.H., Eker L., Aytar A., Daşkapan A., Bayramoğlu M. (2005). Acceptability, reliability, validity and responsiveness of the Turkish version of WOMAC osteoarthritis index. Osteoarthr. Cartil..

[15] Başaran S., Güzel R., Seydaoğlu G., Güler-Uysal F. (2010). Validity, reliability, and comparison of the WOMAC osteoarthritis index and Lequesne algofunctional index in Turkish patients with hip or knee osteoarthritis. Clin. Rheumatol..

[16] Hisli N. (1988). A study on validity and reliability test of the Beck Depression Scale. J. Psychol..

[17] Ulusoy M., Sahin N.H., Erkmen H. (1998). Turkish version of the Beck Anxiety Inventory: Psychometric properties. J. Cogn. Psychother..

[18] Ağargün M.Y., Kara H., Anlar O. (1996). The validity and reliability of the Pittsburgh Sleep Quality Index. Türk Psikiyatr. Derg..

[19] Koçyiğit H., Aydemir Ö., Fişek G., Ölmez N., Memiş A. (1999). Validity and reliability of Turkish version of Short form 36: A study of a patients with romatoid disorder. Drug Treat..

[20] Alkan H., Ardıç F., Erdoğan C., Şahin F., Sarsan A., Fındıkoğlu G. (2013). Turkish version of the painDETECT questionnaire in the assessment of neuropathic pain: A validity and reliability study. Pain Med..

[21] Unal-Cevik I., Sarioglu-Ay S., Evcik D. (2010). A comparison of the DN4 and LANSS questionnaires in the assessment of neuropathic pain: Validity and reliability of the Turkish version of DN4. J. Pain.

[22] Zolio L., Lim K.Y., McKenzie J.E., Yan M.K., Estee M., Hussain S.M., Cicuttini F., Wluka A. (2021). Systematic review and meta-analysis of the prevalence of neuropathic-like pain and/or pain sensitization in people with knee and hip osteoarthritis. Osteoarthr. Cartil..

[23] Yıldırım M.A., Öneş K., Gökşenoğlu G. (2019). Assessment of frequency of neuropathic pain in knee osteoarthritis and its relation to functional state, quality of life and depression. J. Phys. Med. Rehabil. Sci..

[24] Aşkın A., Özkan A., Tosun A., Demirdal U.S., İsnaç F. (2017). Quality of life and functional capacity are adversely affected in osteoarthritis patients with neuropathic pain. Kaohsiung J. Med. Sci..

[25] Ohtori S., Orita S., Yamashita M., Ishikawa T., Ito T., Shigemura T., Nishiyama H., Konno S., Ohta H., Takaso M. (2012). Existence of a neuropathic pain component in patients with osteoarthritis of the knee. Yonsei Med. J..

[26] van Helvoort E.M., Welsing P.M.J., Jansen M.P., Gielis W.P., Loef M., Kloppenburg M., Blanco F., Haugen I.K., Berenbaum F., Bay-Jensen A.-C. (2021). Neuropathic pain in the IMI-APPROACH knee osteoarthritis cohort: Prevalence and phenotyping. RMD Open.

[27] Polat C.S., Doğan A., Özcan D.S., Köseoğlu B.F., Akselim S.K. (2017). Is there a possible neuropathic pain component in knee osteoarthritis?. Arch. Rheumatol..

[28] Oteo-Álvaro Á., Ruiz-Ibán M.A., Miguens X., Stern A., Villoria J., Sánchez-Magro I. (2015). High prevalence of neuropathic pain features in patients with knee osteoarthritis: A cross-sectional study. Pain Pract..

[29] Kubo M., Nosaka Y., Kumagai K., Amano Y., Isoya E., Imai S. (2026). Clinical phenotypes of neuropathic pain and central sensitization in patients with knee osteoarthritis: Demographic data, radiographic severity, and pain intensity. BMC Musculoskelet. Disord..

[30] Finan P.H., Buenaver L.F., Bounds S.C., Hussain S., Park R.J., Haque U.J., Campbell C.M., Haythornthwaite J.A., Edwards R.R., Smith M.T. (2013). Discordance between pain and radiographic severity in knee osteoarthritis: Findings from quantitative sensory testing of central sensitization. Arthritis Rheum..

[31] Argoff C.E. (2007). The coexistence of neuropathic pain, sleep, and psychiatric disorders: A novel treatment approach. Clin. J. Pain.

[32] Manji H.K., Drevets W.C., Charney D.S. (2001). The cellular neurobiology of depression. Nat. Med..

[33] Gonçalves L., Silva R., Pinto-Ribeiro F., Pêgo J.M., Bessa J.M., Pertovaara A., Sousa N., Almeida A. (2008). Neuropathic pain is associated with depressive behavior and induces neuroplasticity in the amygdala of the rat. Exp. Neurol..

[34] Mesci N., Mesci E., Külcü D.G. (2016). Association of neuropathic pain with ultrasonographic measurements of femoral cartilage thickness and clinical parameters in patients with knee osteoarthritis. J. Phys. Ther. Sci..

[35] Hochman J.R., Gagliese L., Davis A.M., Hawker G.A. (2011). Neuropathic pain symptoms in a community knee OA cohort. Osteoarthr. Cartil..

[36] Illeez O.G., Oktay K.N.K., Aktas I., Ozkan F.U., Nazligül T., Begoglu F.A., Kaysin M.Y., Atici A., Akpinar P. (2022). Comparison of the effects of duloxetine and pregabalin on pain and associated factors in patients with knee osteoarthritis. Rev. Assoc. Med. Bras..

[37] Melikoglu M.A., Celik A. (2017). Does neuropathic pain affect the quality of sleep?. Eurasian J. Med..

[38] Guntel M., Huzmeli E.D., Melek I. (2021). Patients with neuropathic pain have poor sleep quality. J. Nerv. Ment. Dis..

